# Falls From Beds Among Elderly Outpatients: Injuries and Outcomes

**DOI:** 10.7759/cureus.57458

**Published:** 2024-04-02

**Authors:** Kyle Nugent, Andrew McCague, Austin Henken-Siefken

**Affiliations:** 1 Surgery, Desert Regional Medical Center, Palm Springs, USA; 2 Trauma and Acute Care Surgery, Desert Regional Medical Center, Palm Springs, USA

**Keywords:** elderly, elderly fall, outpatient falls, fall from bed, falls

## Abstract

Introduction

Falls from beds (FFBs) among outpatient elderly individuals are a prevalent issue, particularly for those aged 65 and above. This presents a notable health challenge with consequences that extend beyond personal well-being, placing a considerable strain on healthcare systems. Fall-related injuries often result in reduced independence, increased morbidity, and, in severe instances, fatalities. It is crucial to address these outpatient falls to safeguard the health and independence of the elderly population.

Methods

This review presents data sourced from a trauma registry covering admissions from March 31, 2016, to December 27, 2021, at Desert Regional Medical Center, a Level 1 Trauma Center in Palm Springs, USA. Over this period, 3,148 patients sought emergency care following falls. The study specifically investigates cases following FFBs, revealing 164 admissions out of the total. Furthermore, it contrasts patient demographics, injury types, and outcomes with existing literature.

Results

This retrospective analysis found that, among the 164 patients admitted to the emergency department over a five-year and eight-month period due to FFBs, 143 were classified as elderly, aged 65 and above. The mean age of those admitted was 76, whereas those not admitted had a mean age of 71. A significant majority, 87%, were hospitalized; within this group, 16% required intensive care. Surgical intervention was necessary for 27 individuals, and there were three fatalities. Soft tissue hematomas were the most common injuries, representing 24% of injuries at admission, closely followed by upper extremity fractures at 21%. Over half of these patients could not return home post-hospitalization, with 41% being transferred to skilled nursing facilities (SNF).

Conclusions

As the aging population in the United States continues to grow, the incidence of falls is on the rise, resulting in injuries like fractures and head trauma. The objectives of this review are to provide an overview of the current literature on FFBs, as well as to emphasize the significant impact of such injuries on the elderly population. Additionally, it includes an analysis of a dataset detailing injuries resulting from bed-related falls, offering a comparison to existing research.

## Introduction

Falls among the elderly are widespread, with a significant proportion of these falls occurring in the bed setting. Globally, falls in people >65 age account for approximately 30% of falls, with approximately 36 million falls being reported annually among the elderly, resulting in over 32,000 deaths [1]. The reported rate of falls among the elderly ranges from 4% to 35% and increases with age, with falls from the bed contributing significantly to these rates [2]. Bed falls can result in moderate to severe injuries like bruises or fractures, impacting an estimated 20-30% of older individuals who experience falls [3]. The exact figures may vary due to different reporting methods and demographic factors, but bed falls are a substantial part of the overall fall burden in the elderly population.

Fractures are the predominant serious injury observed after falls in older adults, with hip fractures being notably common. Approximately 1% of all elderly falls result in hip fractures, which significantly increase the risk of morbidity and mortality post-fall [4]. Furthermore, a study conducted in 2022 over a nine-year period involving 658 residents in a long-term care facility found that falls accounted for over 90% of hip fractures and up to 80% of traumatic brain injuries (TBIs) in older adults [5]. This review seeks to consolidate and encapsulate the latest insights regarding falls that occur in bed among the elderly. Additionally, it intends to provide an overview of bed-fall data collected from a Level 1 Trauma Center over a span of 39 months.

## Materials and methods

This retrospective study was conducted on a deidentified dataset encompassing trauma admissions at Desert Regional Medical Center in Palm Springs, USA, a Level 1 Trauma Center with 368 inpatient beds, spanning from March 31, 2016, to December 27, 2021. The study focused on patients experiencing falls from beds (FFBs), examining patient demographics, injury types, the need for surgical intervention, length of hospital stay, and discharge disposition. Injury classifications included soft tissue injuries, upper and lower extremity fractures, lacerations, TBIs, and hip/femur fractures. Discharge dispositions were categorized into skilled nursing facilities (SNFs), home, unreported cases, hospice, inpatient rehabilitation centers, and morgues. Falls were defined per the World Health Organization's criteria as “an event which results in a person coming to rest inadvertently on the ground or floor or other lower level”. Inclusion criteria comprised patients aged 18 and above who experienced falls while in the outpatient setting, excluding those who fell while admitted as inpatients.

## Results

The data collected encompasses 164 patients treated at Desert Regional Medical Center, a Level 1 trauma facility in Palm Springs, USA, from March 31, 2016, to December 27, 2021, after experiencing outpatient FFBs. The group consisted of 75 males and 89 females, with 143 of them being seniors with an average age of 76. The majority were Hispanic or Latino, making up 68% of the cases. There were 142 hospital admissions, which included 26 instances requiring ICU care (Table 1). Surgical procedures were necessary for 27 individuals, and there were three deaths reported. The patients, on average, stayed in the hospital for 7.3 days, and in the ICU for six days. Patients who were admitted had an average age of 76, while those who were not admitted had an average age of 71 (Table 2). They suffered from a total of 204 injuries categorized into six types; multiple injuries per patient were possible. Specifically, 29 cases involved TBIs, 50 involved soft tissue damages and fractures were numerous with 22 affecting the hip or femur, 42 in the upper limbs, 30 in the lower limbs, and 31 resulted in lacerations (Table 3). Upon discharge, 64 patients went home, 68 to nursing facilities, seven to rehabilitation, and eight to hospice, with four fatalities, and 13 had unspecified discharge destinations (Table 4). A significant percentage of patients with certain injuries did not return home, including 80% with upper limb fractures, 69% with TBIs, and 73% with hip or femur fractures (Table 5).

**Table 1:** Demographic Data ICU: Intensive care unit

Demographic	Number of Patients
Number of patients	164
Gender, n (%)	
Male	75 (46)
Female	89 (54)
Age >65, n (%)	143 (87)
Race, n (%)	
Hispanic or Latino	112 (68)
Not Hispanic or Latino	52 (32)
Admissions, n (%)	142 (87)
ICU admissions, n (%)	26 (16)

**Table 2:** Admission Data ICU: Intensive care unit; LOS: Length of stay; SD: Standard deviation; P: Probability value

Disposition	Number of Patients	Percentage Cases (164)	
Admissions	142	87	
ICU admissions	26	16	
Surgery	27	16	
Death	3	2	
	LOS (days)	SD (days)	
Average LOS of admitted patients	7.3	5.3	
Average LOS in the ICU	6	5.1	
	Average age (years)	SD (years)	P
Admitted	76	20.3	0.28
Not admitted	71	19	

**Table 3:** Presenting Injuries on Admission We have categorized abrasions, contusions, strains, and sprains as soft tissue injuries. Additionally, subdural hematomas, subarachnoid hemorrhages, and concussions have been classified as TBIs. TBI: Traumatic brain injury

Injury	Presented With on Admission
Soft Tissue Injury	50 (24%)
Upper Extremity Fracture	42 (21%)
Laceration	31 (15%)
Lower Extremity Fracture	30 (15%)
TBI	29 (14%)
Hip/Femur Fractures	22 (11%)

**Table 4:** Disposition of Patients From Hospital

Location	Patients
Skilled Nursing Facility (SNF)	68 (41%)
Home	64 (39%)
Unreported	13 (8%)
Hospice	8 (5%)
Inpatient Rehabilitation	7 (4%)
Morgue	4 (2%)

**Table 5:** Type of Injury and Discharge to Home vs. Not Home We have categorized the discharge locations of hospice, SNF, inpatient rehabilitation, and morgue into the category of “not home”. TBI: Traumatic brain injury; SNF: Skilled nursing facilities; P: Probability value

Injury	Home	Not Home	P
Soft Tissue Injury	26 (52%)	21 (48%)	0.005
Upper Extremity Fracture	8 (19%)	34 (80%)	
Laceration	11 (35%)	20 (65%)	
Lower Extremity Fracture	10 (33%)	20 (66%)	
TBI	9 (31%)	20 (69%)	
Hip/Femur Fractures	6 (27%)	16 (73%)	

## Discussion

Falls among the elderly significantly contribute to hospitalizations in the United States. These falls can lead to various levels of injury, ranging from minor scrapes to more serious conditions such as bone breaks, cranial trauma, and in some instances, death [4].

The majority of ED patients following outpatient FFBs were 65 and older. While specific data on the elderly's percentage of FFBs is lacking, the vulnerability of older adults to falls suggests their substantial involvement. Older adults face an increased risk of injuries due to conditions like osteoporosis, osteopenia, Paget’s disease, metabolic bone diseases, and rheumatoid arthritis, contributing to heightened bone fragility. This risk is further amplified by common challenges in the elderly, including diminished vision, weaker muscle strength, and cognitive issues like memory and attention deficits [6]. These factors, coupled with the relatively frequent occurrence of fractures and soft tissue injuries, elucidate the notable rates of hospital and ICU admissions observed in the data. Interestingly, the average age of individuals not admitted was 71, suggesting that younger individuals may have fewer health problems and risk factors that could lead to hospital admission.

Although there is no specific published research on deaths resulting from FFBs, broader studies on fall-related mortality in the elderly highlight its significance. In 2021, 38,742 fall-related deaths occurred among older adults in the United States, with a 41% increase in the age-adjusted fall death rate, rising from 55.3 to 78.0 per 100,000 older adults from 2012 to 2021 [7]. This underscores the considerable impact of falls on the elderly. Our results revealed three patients who experienced fatal outcomes due to FFBs, providing further evidence of the potential severity of such incidents.

In our dataset, we observed that soft tissue injuries were the most common injuries encountered following FFBs. Subsequently, upper extremity fractures became the second most frequently reported injuries. These findings contrast with a 2014 study, which emphasized that fractures were the predominant injuries in older individuals resulting from falls, primarily affecting the wrist, arm, ankle, and hip [6]. The prevalence of upper extremity fractures in these cases is likely due to the phenomenon known as falling on an outstretched hand (FOOSH). When a person falls, their innate reflex is to extend their arms to cushion the impact, thereby increasing the likelihood of landing on their hands and wrists. This instinctive response can result in fractures occurring in the upper extremities, with the wrists and forearms being particularly susceptible to such injuries [8].

Hip fractures, in particular, are notably concerning for the elderly due to their profound long-term effects. Such injuries drastically affect seniors' mobility and self-sufficiency and are often associated with a range of severe health issues. These include sustained immobility, an escalated risk of future falls, and an increased probability of needing long-term care [9]. Recovery from hip fractures in older adults is often protracted and difficult, requiring comprehensive rehabilitation and may result in a lasting decline in life quality.

After fractures, soft tissue hematomas, lacerations, pain, head injuries, sprains, and strains are injuries that are also commonly seen in the elderly after bed falls [6]. Soft tissue hematomas, often occurring in areas like the elbows and knees, result from the impact of the fall causing blood vessels under the skin to rupture. Lacerations are also frequent, particularly on the elbow, forearm, and hands, where the skin may tear or cut upon contact with hard surfaces or sharp objects during the fall. Pain is another typical outcome of falls, commonly felt in various body parts, especially the torso, and can be attributed to muscle strains, bruises, or other internal injuries. Head injuries, which can range from concussions to more severe TBIs, are especially serious in the elderly due to their potential long-term consequences. Additionally, sprains and strains are often seen, resulting from sudden and awkward movements during a fall, affecting ligaments and muscles [6]. Our findings suggest that TBIs and lacerations are injuries that are relatively infrequent in hospital admissions after FFBs. The lower height of beds typically does not pose a substantial risk for severe head injuries, and the usual absence of sharp objects near beds lessens the likelihood of lacerations occurring. It is important to note that these specific findings pertain to incidents involving bed falls, as opposed to falls in a broader context where TBIs and lacerations may be more prevalent. Concerning all etiologies of falls in the elderly, TBIs are significant, constituting 32% of hospital admissions and over half of the mortality associated with such incidents [10].

The duration of hospitalization after a FFB can vary depending on the severity of injuries and the individual's overall health. A study focused on inpatient falls and orthopedic injuries in elderly patients reported an average length of stay (LOS) of 2.4 weeks following a fall [11]. It's essential to highlight that this study specifically concentrated on orthopedic injuries within an inpatient population, not encompassing all injury types in an outpatient setting. In our dataset, the average LOS stands at 7.3 days, markedly shorter than the previously mentioned inpatient study [11]. This disparity may be attributed to the relatively milder and more common injuries observed in our dataset, primarily soft tissue bruising.

It is noteworthy that a significant portion of the patients in our dataset did not go back to their homes following hospitalization. Instead, the majority were discharged to SNF, hospice care, or inpatient rehabilitation centers to receive ongoing care. This discovery highlights the considerable severity of injuries frequently sustained by elderly individuals after FFBs, necessitating extensive post-hospitalization medical attention and rehabilitation. The decision of where to discharge a patient depends on several variables: the fall's severity, the patient’s functional and cognitive abilities, and whether they have support available at home. While specific discharge data for elderly patients post-fall are not published, general guidelines suggest that patients with minor injuries and a strong support system may go home, with or without home health services. In contrast, those with more severe injuries or lacking home support are often moved to an SNF or similar care facilities for the necessary support and rehabilitation [12]. It is also worth noting that our data revealed a significant trend. Patients with severe injuries such as fractures, TBIs, and lacerations were more frequently discharged to SNFs, hospice care, and inpatient rehabilitation. These injuries necessitated specialized and intensive care due to their severity. In contrast, milder soft tissue injuries predominantly resulted in patients being discharged home.

This retrospective study conducted using data from a single hospital faces several limitations and biases. Firstly, the relatively small sample size could restrict the ability to identify rare events or conditions that might be more apparent in larger datasets. Additionally, the study's reliance on data from a single hospital limits its generalizability, as findings may not be universally applicable due to the specific patient population and healthcare setting represented. Furthermore, the study's timeframe, spanning from 2016 to 2021, introduces the potential for time-related bias. Changes in diagnostic criteria or treatment practices over this period could influence the study's results, impacting the interpretation and validity of findings.

## Conclusions

Falls, especially those occurring from beds, represent a major cause of injury among the elderly. Our study revealed that among elderly individuals who experienced falls, soft tissue injuries and upper extremity fractures were frequently encountered. In contrast, TBIs and hip fractures, although less common, imposed a substantially greater burden. This elevated injury burden results in a heightened prevalence of elderly patients being discharged to post-hospital care settings, including SNFs, hospice care, or inpatient rehabilitation centers.
